# TGF-β orchestrates the phenotype and function of monocytic myeloid-derived suppressor cells in colorectal cancer

**DOI:** 10.1007/s00262-021-03081-5

**Published:** 2021-11-02

**Authors:** Luciana Gneo, Nagy Rizkalla, Rahul Hejmadi, Francis Mussai, Carmela de Santo, Gary Middleton

**Affiliations:** 1 Institute of Immunology and Immunotherapy, University of Birmingham, Edgbaston, Birmingham, B152TT UK; 2 Department of Pathology, University Hospital Birmingham, Birmingham, UK

**Keywords:** M-MDSCs, TGFβ, IL-10, Colorectal cancer, pSTAT3

## Abstract

**Background:**

Monocytic myeloid-derived suppressor cells (M-MDSCs) are significantly expanded in the blood of colorectal cancer (CRC) patients. However, their presence and underlying mechanisms in the tumour microenvironment of CRC have not been examined in detail.

**Methods:**

Tumour tissues and peripheral blood from CRC patients were analysed for the presence of M-MDSCs. The mechanisms of suppression were analysed by blocking pathways by which MDSCs abrogate T cell proliferation. Co-culture of CRC cells with monocytes were performed with and without cytokine blocking antibodies to determine the mechanism by which CRC cells polarise monocytes. Multi-spectral IHC was used to demonstrate the intra-tumoral location of M-MDSCs.

**Results:**

Tumour tissues and blood of CRC patients contain M-MDSCs which inhibit T cell proliferation. Whilst inhibition of arginase and nitric oxide synthase 2 fail to rescue T cell proliferation, blockade of IL-10 released by these HLA-DR^−^ cells abrogates the suppresivity of M-MDSCs. Tumour conditioned media (TCM) significantly reduces HLA-DR expression, increases IL-10 release from monocytes and causes them to become suppressive. TGF-β is highly expressed in the TCM and accumulates in the plasma. TGF-β reduces HLA-DR expression and drives monocyte immunosuppressivity. The invasive margin of CRC is enriched in CD14^+^ HLA-DR^−^ cells in close proximity to T cells.

**Conclusions:**

Our study demonstrates the cross-talk between CRC cells, M-MDSCs and T cells. Characterisation of CRC M-MDSCs point to therapeutic avenues to target these cells in addition to TGF-β blockade.

**Supplementary Information:**

The online version contains supplementary material available at 10.1007/s00262-021-03081-5.

## Background

The identification of a cell as a myeloid-derived suppressor cell (MDSC) requires both the appropriate phenotypic characterisation and evaluation of functional characteristics [1]. In humans three main MDSC phenotypes are recognised: polymorphonuclear MDSCs (PMN-MDSCs: CD15^+^ CD11b^+^ CD14^−^), monocytic MDSCs (M-MDSCs: CD14^+^ CD11b^+^ CD15^−^ HLA-DR ^low/−^) and early-stage MDSCs (eMDSCs: Lin^−^ (CD3/14/15/19/56) HLA-DR^−^ CD33^+^). Accepted functional verification assays include autologous systems (typically the suppression by the added MDSC populations of CD3/CD28 induced T cell proliferation) and/or allogeneic systems (suppression of proliferation in allogeneic mixed lymphocyte reactions (MLR)).

In the UK alone, there are over 42,000 new cases of colorectal cancer (CRC) per annum and over 16,000 people die of the disease each year making it the second commonest cause of cancer death [2]. The therapy of metastatic proficient mismatch repair CRC has remained largely unchanged in the last 10 years. Whilst checkpoint blockade has become a key therapeutic modality in advanced deficient mismatch repair CRC such therapy has been disappointing in proficient mismatch repair. Whilst anti-MDSC strategies have enhanced the efficacy of checkpoint blockade in pre-clinical models [3, 4] whether tumoral MDSCs are important in limiting checkpoint blockade efficacy in human CRC is currently unknown. However, an accurate understanding of their biology would be of value in informing the rational design of combinatorial immunotherapy in CRC. Although there are several studies on MDSCs in CRC, many of the studies provide limited data on tumoral MDSCs [5–7] and/or lack functional readouts [8–12]. One study that phenotypically and functionally analysed tumoral MDSCs in CRC focussed exclusively on PMN-MDSCs [13] Accumulation of CD66b + PMN-MDSCs appeared to be due to enhanced migration related to IL-8 and GM-CSF produced by γδT17 cells. However, not all intra-tumoral CD66b + cells in CRC are suppressive; tumour-associated CD66b + neutrophils CRC tissues activate T cells and stimulate IFNγ production [14]. Further, the principal IL-17 producing cells in CRC appear not to be γδT17 but Th17 cells: γδT cells are mainly IL-17 negative [15].

We decided to focus on M-MDSCs in CRC for a number of reasons besides this conflicting data on tissue CD66b^+^ cells. Concerns have been raised about the functional impact of the manipulation of dissociated tissue on PMN-MDSCs and the limitations of the commonly used density centrifugation to isolate circulating PMN-MDSC [16, 17]. Some studies in mice have clearly shown that whilst PMN-MDSCs have minimal suppressive effects on antigen stimulated CD8 + T cells and are non-tolerogenic in vivo, M-MDSCs are highly immunosuppressive [18, 19]. Furthermore, M-MDSCs appear to be important precursors of suppressive tumour-associated macrophages [20]. Macrophages derived from M-MDSCs have higher amounts of S100A9, nitric oxide synthase 2, ARG1 and much lower HLA-DR expression than those derived from classical monocytes, are polarised toward an M2 phenotype and are highly immunosuppressive. Finally, M-MDSCs appear to be the main immunosuppressive MDSC population in CRC [21]. Whilst M-MDSCs are significantly expanded in the blood of CRC patients the difference in PMN-MDSCs is non-significant. The abundance of M-MDSCs doubled on treatment suggestive of M-MDSCs mediating therapy resistance. Higher M-MDSC levels portend a worse progression free survival to first-line oxaliplatin-based therapy [5]. Whilst peripheral M-MDSCs were shown to suppress proliferation [21] limited mechanistic work was performed and no analysis of intra-tumoral CRC M-MDSCs was undertaken. Here, we demonstrate the mechanism by which CRC cells polarise monocytes to become M-MDSCs and the mechanism by which these cells both in blood and tumour suppress T cell proliferation, thus significantly advancing our knowledge of M-MDSCs in CRC. Furthermore, these data can be used to support translation into the clinic of anti-M-MDSC strategies in order to potentially enhance the effectiveness of immunotherapy in CRC.

## Materials and methods

### Blood and tumour sample collection and patient inclusion and exclusion criteria

Heparinized blood samples were obtained from patients with histologically confirmed colon carcinoma (*n* = 67) prior to surgery, following informed consent, at the University of Birmingham Hospitals Trust, UK. Fresh tumour samples (*n* = 41) or matched adjacent normal intestinal tissue (*n* = 27) from diagnostic surgery were collected and processed within 12 h. Blood was also obtained from age-matched healthy donors at the University of Birmingham, UK (*n* = 33). There was no lower or upper age limit for participation and both males and females were included. The only exclusion criteria that were used for patient sample collection were cancers in those patients undergoing emergency surgery and/or cancers arising on the background of known inflammatory bowel disease. Participants did not undergo randomization as this was irrelevant to our study.

### Flow cytometry analysis of whole blood and tumours

Red blood cells were lysed using ammonium chloride solution according to manufacturer’s instructions (Qiagen, Cat# 1,045,722) for 10 min at room temperature prior to antibody staining. Tumour and tissue samples were digested using Type II collagenase (Sigma; Cat#C6885-100MG;) for three hours at 37 °C, washed and re-suspended in RPMI + 10% FBS; R10% (RPMI-1640 (Sigma-Aldrich; Cat# R8758) with 10% heat-inactivated fetal bovine serum, glutamine (1X), sodium pyruvate (1X) and Penicillin–Streptomycin (RPMI 10% = R10%). Immune populations were identified by staining with anti-human antibodies: anti-CD33-APC (BioLegend Cat# 303,408, RRID:AB_314352), anti-HLA-DR-PE (BD Biosciences Cat# 555,812, RRID:AB_396146), anti-CD14-PE-Cyanine7 (Thermo Fisher Scientific Cat# 25-0149-42, RRID:AB_1582276), anti-CD68-Alexa Fluor 488 (BioLegend Cat# 333,812, RRID:AB_2074832), anti-CD163-Brilliant Violet 421 (BioLegend Cat# 333,612, RRID:AB_2562463), anti-CD206-APC/Cyanine7 (BioLegend Cat# 321,120, RRID:AB_2144930) and anti-CD15-FITC (BioLegend Cat# 301,904, RRID:AB_314196) antibodies on ice for 30 min in Flow Buffer consisting of 1X PBS (Sigma-Aldrich: P3813) and 2%BSA (Sigma- Aldrich: 9048-46-8). Cells were acquired using a Cyan and CytoFLEX flow cytometers and analysed using FlowJo and CytoExpert2.2.

### Multi-spectral immunohistochemistry and multiplex staining

Colorectal carcinoma sections, from diagnostic tumour biopsies (*n* = 5), were deparaffinised in Histoclear (National diagnostics) and ethanol, and rehydrated in 0.3% hydrogen peroxide for 15 min. Antigen retrieval was performed in 10 mM sodium citrate buffer (pH 6.0) for 20 min in a microwave oven. Slides were cooled and washed prior to blocking in 5X caesin (Thermofisher) for 30 min at room temperature. Multi-colour immunofluorescent staining was carried out on the Leica Bond Max using an Opal™ 7-colour fIHC kit (Perkin Elmer Cat# NEL80100KT) and the following primary antibody fluorochrome combinations: CD3/Opal 520, CD14/Opal 570, HLA-DR/Opal 620 and CK (monoclonal mouse anti-human cytokeratin (concentrate) antibody clone AE1/AE3)/Opal 650. Staining was imaged with the Vectra Automated Quantitative Pathology Imaging System (Perkin Elmer) and 7–11 representative fields per case quantitatively analysed using Inform Advanced Image Analysis software v 2.3 (Perkin Elmer). Nearest Neighbour analysis performed using the *R* programming language Version 3.5.1(*R* Core Team (2018) https://www.R-project.org/.) in conjunction with the inForm Helper Functions *R* package Version 0.1.2. (https://akoyabio.github.io/phenoptr/) and 'Tidyverse' *R* package Version 1.2.1. (https://CRAN.R-project.org/package=tidyverse).

### Blood and tumour cell isolation and sorting

Peripheral blood mononuclear cells (PBMC) were isolated from the whole blood of patients or healthy donors following Lymphoprep (Stem Cell Technologies, Cat# 07,851/07861) centrifugation. The CD14^+^ PBMC fraction was isolated by incubation with anti-CD14 microbeads (Miltenyi Biotech; Cat# 130-050-201) and sorting using MACS LS separation columns (Miltenyi Biotech, Cat# 130-042-401) according to manufacturer’s instructions. Where indicated CD14^+^ cells were then further isolated into HLA-DR^+^ and HLA-DR^−^ fractions by a second sorting with HLA-DR microbeads (Miltenyi Biotech, Cat# 130-046-101) and LS column separation. Cell purity was > 98% as confirmed by flow cytometry using anti-CD14-PE-Cyanine7, anti-HLA-DR-PE or anti-CD3 (BD Biosciences Cat# 555,335, RRID:AB_398591) antibodies.

The CD14^−^ leukocyte population from healthy donors was used as responding T cells in the anti-CD3/CD28 antibody proliferation assays. Cell populations were similarly isolated from the collagenase digested tumours suspension using anti-CD14 MACS beads (CD14^+^ myeloid cells) or anti-CD326 EPCAM MACS beads (Miltenyi Biotech; Cat# 130-061-101)(CD326^+^ tumour cells). Purity was confirmed by flow cytometry. No effect of collagenase on cell surface marker expression or cell viability was observed.

### Cell line authentication and validation and monocyte polarisation with TCM and cytokines

To generate tumour conditioned media (TCM), CD326^+^ sorted patients’ tumour cells were first plated (1 × 10^6^ cells) and cultured for 48 h. The conditioned media was removed and filtered prior to use. Colorectal cancer cell lines HT29 (ATCC^R^ HTB-38™), HT55 (ATCC^R^ HTB-55™), and WiDr (ATCC^R^ CCl-218™) were all freshly obtained from the ATCC collection to ensure full authentication and validation and tested negative for mycoplasma contamination and were similarly cultured in RPMI + 10% FBS, with penicillin–streptomycin. Sorted healthy donor CD14^+^ monocytes were plated in R10% or with 100% of total volume TCM or 10 ng/ml of human recombinant cytokines (PeproTech: TGF-β Cat# 100–21-50; IL-6 Cat# 200–06; IL-10 Cat# 200–10; G-CSF Cat# 500-P43; GM-CSF Cat# 300–03 and VEGF Cat# 100–20) in 24 well plates for 48 h. These polarised monocytes were harvested, washed twice, and used as described below. Where indicated CD14^+^ cells (1 × 10^6^ cells) were pre-treated for 1 h with TGF-β receptor inhibitor (LY364947 [5 µM]; TOCRIS Biotechne) followed culture in TCM or TGF-β (10 ng/ml) for 48 h.

### T cell proliferation assay

T cells (2 × 10^5^) were cultured in 96 well flat bottom plates coated with anti-CD3 antibody (3 μg/ml) ((eBioscience, Thermo Fisher Scientific Cat# 16-0037-85, RRID:AB_468855)) and anti-CD28 antibody (2 μg/ml) (Thermo Fisher Scientific Cat# 16-0289-85, RRID:AB_468927), in 200 μl R10%. The suppressive ability of sorted CD14^+^ cells from patients or from healthy donors following polarisation was assessed by co-culturing purified cells together with allogeneic T cells. Where indicated anti-IL-10 Receptor Antibody (10 μg/ml) ((Thermo Fisher Scientific Cat# 16-7108-85, RRID:AB_469229) (Isotype control, Rat IgG Ƙ Isotype, (Thermo Fisher Scientific Cat# 16-4301-85, RRID:AB_470154) were added to cultures to inhibit IL-10 binding. Cells were incubated at 37 °C, 5% CO2 for 4 days and then 1 μCi/well ^3^H-thymidine (Perkin Elmer Life Sciences, Beaconsfield, UK) was added for 12–16 h. ^3^H-thymidine incorporation was measured using a TopCount reader (Perkin Elmer). Data are expressed as a percentage of T cell proliferation driven by antibody co-stimulation in the presence of CD14^+^ cells, compared to T cell proliferation in the absence of suppressive cells (100%).

### ELISA

The concentration of cytokines within conditioned media of patient-derived CD14^+^ cells or TCM polarised healthy donor CD14^+^ or plasma was assessed using a LEGENDplex Multi-Analyte Flow Assay according to the manufacturers’ instructions (LEGENDplex Human Proinflammation Panel 1 (13-plex) w/VbP; Biolegend; Cat# 740,003, LEGENDplex HU Th Cytokine Panel 1 (13-plex) w/VbP; BioLegend Cat#740,721, LEGENDplex™ HU Neuroinflammation Panel 1 (13-plex) w/VbP, BioLegend; Cat#740,796).

### Western blotting

Cells were extracted in lysis buffer (RIPA Buffer 1X (Thermo Scientific, Cat# 89,900) containing PhosSTOP 1X (Roche, Cat# 04,906,845,001) and Complete Inhibitor 1X (Roche, Cat# 64,693,132,001)) and protein concentrations were assessed by Bradford protein concentration assay (Sigma-Aldrich, Cat# 23,236). Samples were mixed with reducing SDS-PAGE sample buffer (4X Lamelli Sample (BioRad, Cat#1,610,747) + 10% β-Mercaptoethanol) and subjected to Western Blotting. Briefly, proteins separated by reducing SDS-PAGE, were trans-blotted onto Hybond C^+^ nitrocellulose membranes by electrophoresis. The membranes were incubated for 1 h with blocking solution (5% BSA in PBS-T(Tween 1%)) to block the non-specific protein binding-site prior to incubation with primary antibodies, at recommended dilutions, for 16 h (ON) at 4 °C. Membranes were then washed in PBS-T, incubated with secondary HRP-conjugated antibodies diluted in blocking solution and immuno-reactive species detected by chemiluminescence reaction (BioRad, Cat#170-5061), according to manufacturer’s instructions. The following antibodies has been used: Phospho-Smad2(Ser465/467)/Smad3(Ser423/425)(D27F4)(Cell Signalling Technology Cat# 8828S, RRID:AB_2631089; Rabbit), Anti-SMAD2/3 (Cell Signalling Technology Cat# 5678S, RRID:AB_10693547; Rabbit), Phospho-STAT3(Tyr705) (D3A7) (Cell Signalling Technology Cat# 9145S, RRID:AB_2491009; Rabbit), Anti-STA3(D1B2J) Cell Signalling Technology Cat# 30,835, RRID:AB_2798995; Rabbit), Phospho-p38 MAPK(Thr180/Tyr182) (3D7) (Cell Signalling Technology Cat# 9215S, RRID:AB_331762; Rabbit), Anti-p-38 MAPK (Cell Signalling Technology Cat# 9212S, RRID:AB_330713; Rabbit), Phospho-p44/42 MAPK (Cell Signalling Technology Cat# 9101S, RRID:AB_331646; Rabbit), p-44/42 MARK (Cell Signalling Technology Cat# 9102S, RRID:AB_330744; Rabbit),β-Actin (Cell Signalling Technology Cat# 4970, RRID:AB_2223172; Rabbit); HRP-conjugated anti-rabbit IgG (Cell Signalling Technology Cat# 7074S, RRID:AB_2099233).

### Statistical analysis

All the data were analysed using Graph Pad Software (RRID-SCR_002798. A T test analysis was used to determine the statistical significance of the difference in unpaired and paired groups according with the experimental design. A *p* values < 0.05 were considered to represent statistically significant events. A linear regression analysis was used to quantify the relationship between two variables, using a 95% confidence interval.

### Study approval

In accordance with the Declaration of Helsinki, patient samples were obtained after written, informed consent prior to inclusion in the study. Regional Ethics Committee (REC Number 10/H0501/39) and local hospital trust research approval for the study were granted for the University of Birmingham Hospitals Trust.

## Results

### Suppressive CD14^+^ cells are elevated in the tumours of CRC patients

**Fig. 1:**
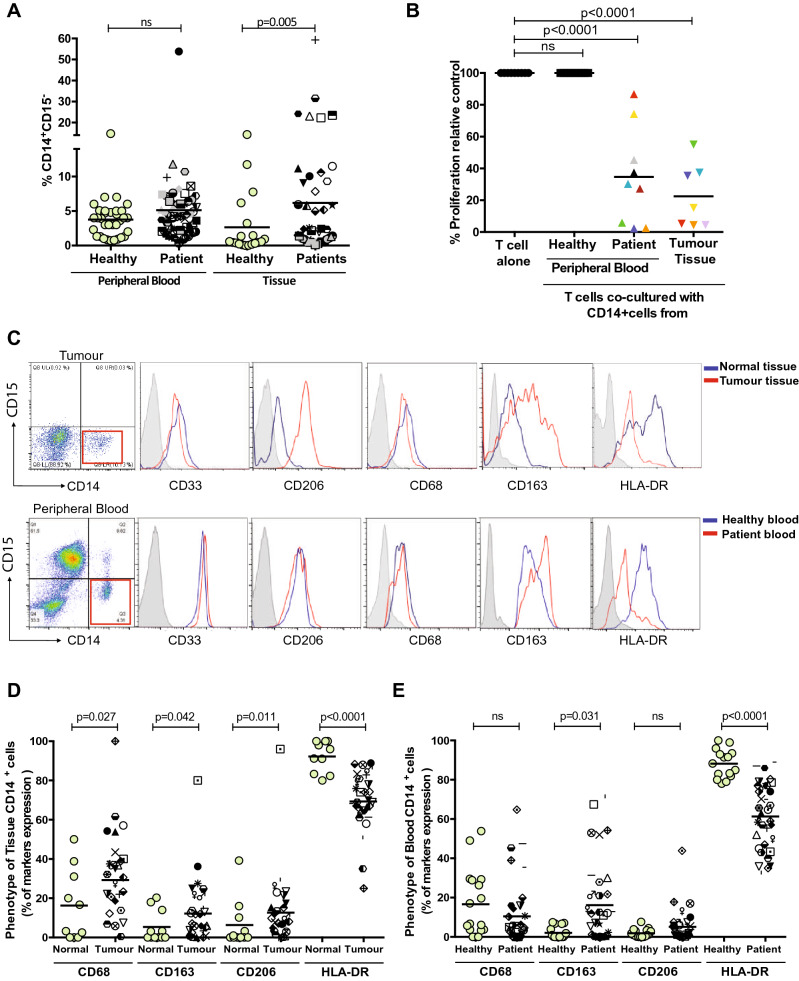
CD14+ cells in the blood and tumour suppress peripheral blood leukocyte proliferation in CRC patients **a** Increased frequency of CD14+ monocytes staining in the peripheral blood (n=53) and tissue (n=18) of colorectal cancer samples and healthy control (*p*=0.005). **b** Allogeneic T cell proliferation under anti-CD3/CD28 antibody stimulation is suppressed with the addition of CD14+ cells from tissue (n=7) and the blood (n=9) of CRC patients, as measured by 3H-Tymidine uptake, compared to CD14+cells from healthy donors (*p*<0.0001). **c** Phenotypic analysis of CD14+ cells from blood and tissue of representative CRC patient. Flow analysis of CD14+ cells shows an increased expression of CD206,CD68, CD163 markers in the tumour tissue **d** and not in the blood **e** of CRC patients, instead and a significant down regulation of HLA-DR expression is measured in both the compartments compared healthy control

Although the presence of granulocytic MDSCs (G-MDSCs) have been well defined in colorectal cancer patients [14], the presence of monocytic MDSCs (M-MDSCs) within the tumour microenvironment is less clear. Dissociation of human colorectal cancers revealed a significant increase in the frequency of CD14^+^ cells in the tumour compared to matched non-cancerous tissue (*p* = 0.005; Fig. 1a). There was no increase in the proportion of circulating CD14^+^ cells in the blood of patients compared to healthy donors (Fig. 1a). To examine the effect of these myeloid cells on T cells co-culture proliferation assays were performed. Sorted CD14^+^ cells from colorectal tumours and from the peripheral blood strongly suppressed T cell proliferation (*p* < 0.0001, Fig. 1b). Healthy donor blood CD14^+^ cells had no effect on T cell proliferation (Fig. 1b).

### Tumoral and blood CD14^+^ HLA-DR^−^ cells are M-MDSCs and suppress T cell proliferation via IL-10 production

The identification of immunosuppressive CD14^+^ cells in the blood and tumour of CRC patient raised the question as to whether these cells could be defined as M-MDSCs, which are characterized by reduced HLA-DR expression. The immunophenotype of CD14^+^ cells of CRC patients compared to healthy counterparts, show equivalent expression of myeloid marker CD33 (Supp Fig. 1A). CD14^+^ cells from tumour tissue have higher expression of the macrophage markers CD68, CD206 and CD163 (Fig. 1c, d) but only CD163 was increased in patient blood CD14^+^ cells (Fig. 1c, e). There was significant down-regulation of HLA-DR expression in both the blood and tissue of CRC patients (Fig. 1c–e). There was no correlation between the frequency of CD14^+^HLA-DR^−^ cells in the blood and tumours of patients (Supp Fig. 1B).

**Fig. 2:**
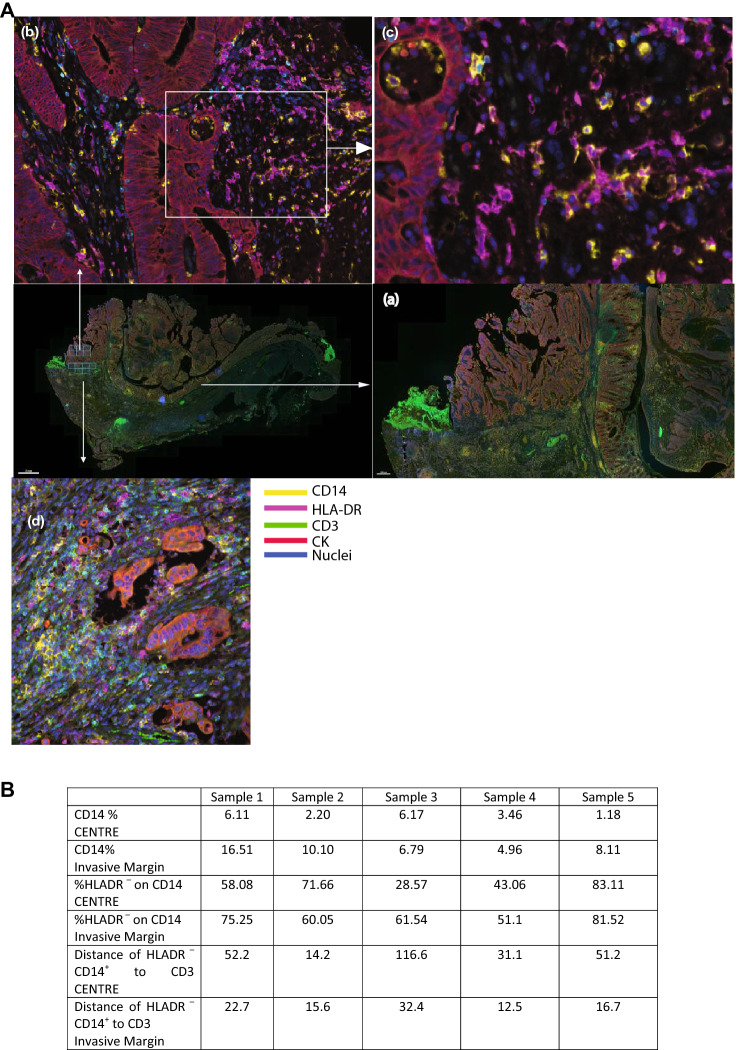
Tumour localization of CD14+ myeloid cells in colorectal cancer. A) Representative multispectral immunohistochemical staining of colorectal cancer section. The squares in the middle right panel and magnified in (a) indicates the area of colon analysed with higher magnification where arrows indicate an increase in magnification of the specified area - upper squares, centre of tumour and lower squares, invasive margin: the magnified invasive margin image is shown in (d) and those of the tumour centre are shown in (b and c). CD14: Yellow; HLA-DR: Magenta; CD3: Cyan/green; CK:Red; nuclei: Blue. B) Frequency of CD14+ cells as a percentage of total cells in colorectal cancer tissue at the tumour centre and invasive margins, the percentage of DR–CD14+ cells and the distance of DR–CD14+ cells to CD3+ cells as determined by VECTRA spectral analysis (n=5)

To identify the geographical distribution of CD14 + cells, the intra-tumoral microenvironment was examined in-situ using multi-spectral immunohistochemistry (Fig. 2 and Supp Fig. 2). Staining identified that the majority of CD14^+^ cells resided as a layer spanning the invasive margin of the tumours (yellow staining Fig. 2a panel (a) and Supp Fig. 2), and that the majority of these cells are HLA-DR^−^ confirming the flow cytometry findings (Fig. 2a panel (b)). In contrast the tumour centres have relatively lower numbers of infiltrating CD14^+^ cells (Fig. 2a panel (b) and (c)). The CD14^+^HLA-DR^−^ cells separate the tumour cells (red staining Fig. 2a panel (d)) from the CD3^+^ T cells (green staining Fig. 2a panel (d)), such that the majority of the CD3^+^ T cells are furthest from the malignant cells (Fig. 2b). Very few T cells infiltrate the tumour centre. Nearest neighbour analysis demonstrated that the median distance from the CD14^+^ HLA-DR^−^ cells to CD3^+^ cells at the invasive margin was either much less (Samples 1,3,4 and 5) or the same (Sample 2) as the distances from the CD14^+^ HLA-DR ^−^ cells to the CD3^+^ cells in the centre of the tumour. Thus, the invasive margin of colorectal cancers are enriched in CD14^+^ HLA-DR ^−^ cells in close proximity to T cells.

**Fig. 3:**
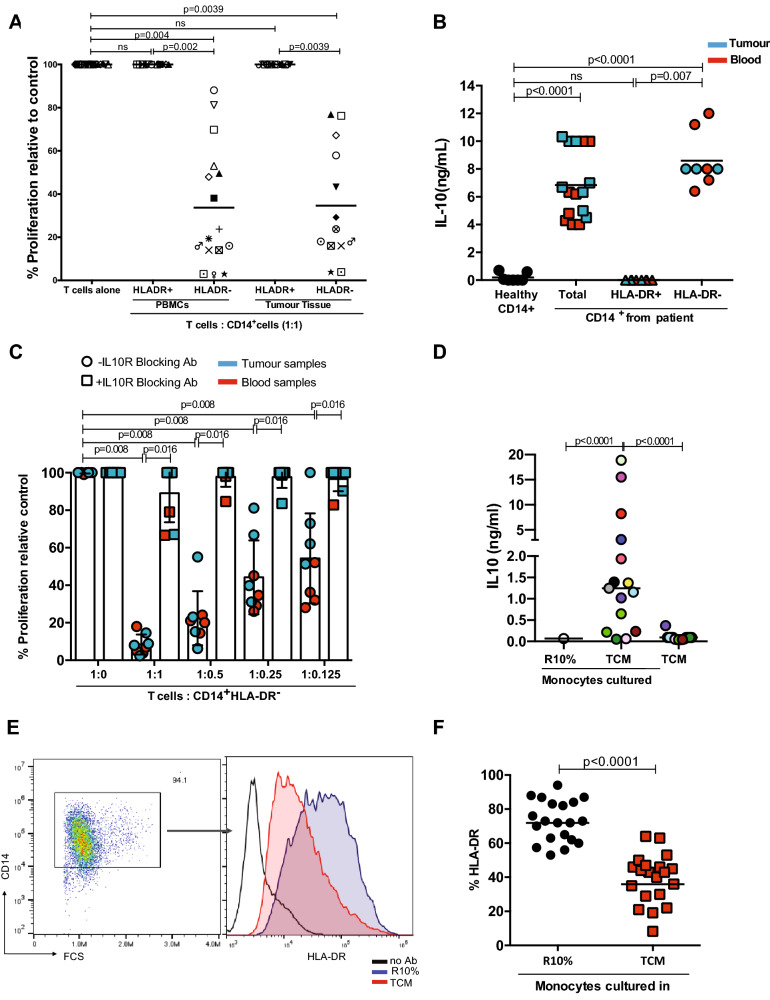
CD14+HLA-DR- cells are the major immunosuppressive cells in CRC. **a** Allogeneic T cell proliferation under anti-CD3/CD28 antibody stimulation is suppressed with the addition of CD14+HLA-DR- cells from the blood (n=16) and the tissue (n=13) of CRC patients, as measured by 3HTymidine uptake. The CD14+HLA-DR+ population is not suppressive. The 1:1 ratio of T cells:CD14+HLA-DR- cells is shown. Each colour represents a CRC patient. **b** Significantly higher level of IL-10 was detected by ELISA in the supernatant of CD14+ (blood samples n=8, tumour tissue n=8)and CD14+HLA-DR- cells (blood samples n=5, tumour tissue n=3) compared healthy control. **c** IL-10 receptor blocking antibody (10μg/ml) inhibits the suppressive activity of CD14+HLA-DR- cells of CRC patients restoring T cell proliferation (n=10). **d** Colorectal tumour conditioned media (TCMs, n=15)drive the release of IL10 from healthy monocytes after 48h incubation. **e** A representative histogram of HLA-DR expression on monocytes after 48h incubation with TCM. **f** Down regulation of HLA-DR on monocytes cultured with 20 TCMs

To formally demonstrate that the M-MDSC population resided in the HLA-DR^−^ fraction, CD14^+^ cells were sorted according to HLA-DR status and T cell proliferation assays performed. For both blood and cancer tissues the HLA-DR^−^ cells suppressed anti-CD3/CD28 stimulated T cells (Fig. 3a and Supp Fig. 3a, b). However, the HLA-DR^+^ cells had no suppressive effect (Fig. 3a and Supp Fig. 3A and 3B).

M-MDSCs have been reported to suppress T cell proliferation by a number of mechanisms. The effect of inhibiting arginase 1 (ARG1) and nitric oxide synthase 2 was assessed using the small molecule inhibitors NOHA and L-NMMA, respectively, and was found not to rescue T cell proliferation inhibited by colorectal cancer patient’s CD14 + cells (Supp Fig. 3C). MDSCs may also suppress T cell responses through cytokine release. Previous studies have shown that in ovarian cancer CD14^+^ cells can suppress T cell proliferation via IL-10 [22]. To identify which cytokines were released, sorted cells from patients’ tumours and blood were placed in culture and supernatants tested by ELISA. CD14^+^ cells from both tumours and blood released significantly more IL-10 (Fig. 3b) than those from healthy donors and HLA-DR^−^ cells from colorectal patients are the main source of IL10 secretion (Fig. 3b).To determine whether the suppressive activity of the M-MDSCs in both tumours and blood might be IL-10 dependent, IL-10 receptor blocking antibody was added to T cell proliferation assays. Blockade of IL-10 signalling led to restoration of T cell proliferation in the presence of M-MDSCs from both the blood and tumours (Fig. 3c) of colorectal cancer patients. Thus, CRC M-MDSCs in CRC suppress T cell proliferation via IL-10 release.

### Colorectal cancer cells polarize tumour-associated monocytes to a M-MDSC phenotype

**Fig. 4:**
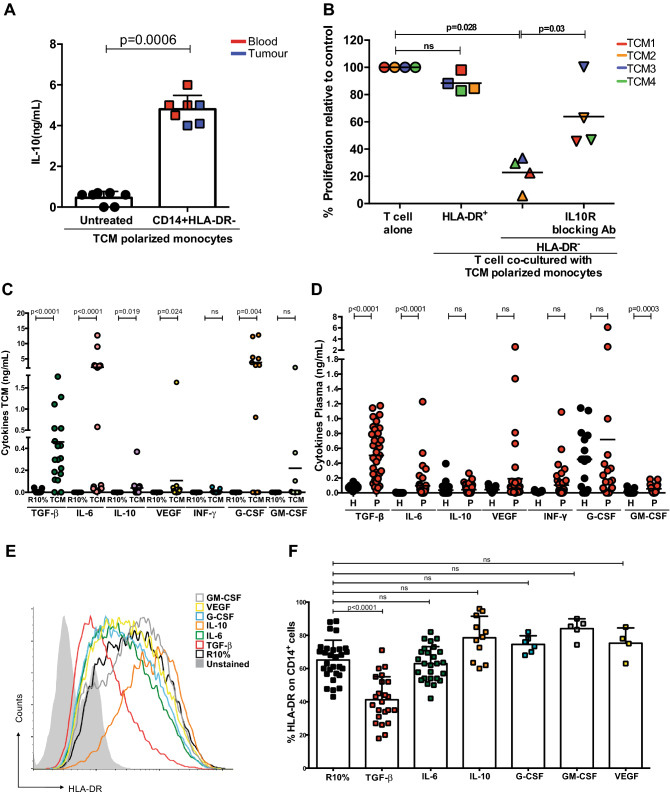
Colorectal tumour conditioned media drives IL10 secretion. **a** Significantly higher level of IL-10 was detected by ELISA in the supernatant of CD14+HLA-DR- cells polarized by TCM (blood samples n=4, tumour tissue n=3) compared healthy control. **b** IL-10 receptor blocking antibody (10μg/ml) inhibits the suppressive activity of TCMs polarized CD14+HLA-DR- cells restoring T cell proliferation (n=4). Higher concentration of TGF-β was measured by ELISA in TCM (n=19) **c** and in the plasma **d** of colorectal patients (n=39 and healthy plasma n=10). **e** A representative histogram of HLA-DR expression on monocytes cultured with cytokines for 48h. **f** Down regulation of HLA-DR on monocytes cultured with cytokines for 48h was observed by Flow analysis (n=23 TGFβ, n=26 IL-6, n=11 IL-10, n=6 G-CSF, n=5 GM-CSF and n=4 VEGF)

Malignant cells from fresh patients’ tumours were isolated by sorting for CD326(EPCAM) and placed in culture to generate tumour conditioned media. Tumour conditioned media (TCM) conferred on CD14^+^ cells from healthy donors the ability to release IL-10, whilst complete media had no effect (Fig. 3d). Furthermore, whilst incubation in complete media had no effect on the level of HLA-DR expression on healthy donor monocytes TCM from primary tumour cells and colon cell lines significantly reduced HLA-DR expression (Fig. 3e, f and Supp Fig. 4A). TCM significantly enhanced IL-10 production from the HLA-DR^−^ fraction (Fig. 4a and Supp Fig. 4B). Blockade with anti-IL-10 receptor antibody restored T cell proliferation to different extents that had been suppressed by TCM polarized CD14^+^ cells (Fig. 4b).Thus, the TCM of colorectal cancer cells reduces monocyte HLA-DR expression and up-regulates IL-10 production which drives a T cell suppressive phenotype.

### TGF-β from colorectal tumour cells drives M-MDSC generation

**Fig. 5:**
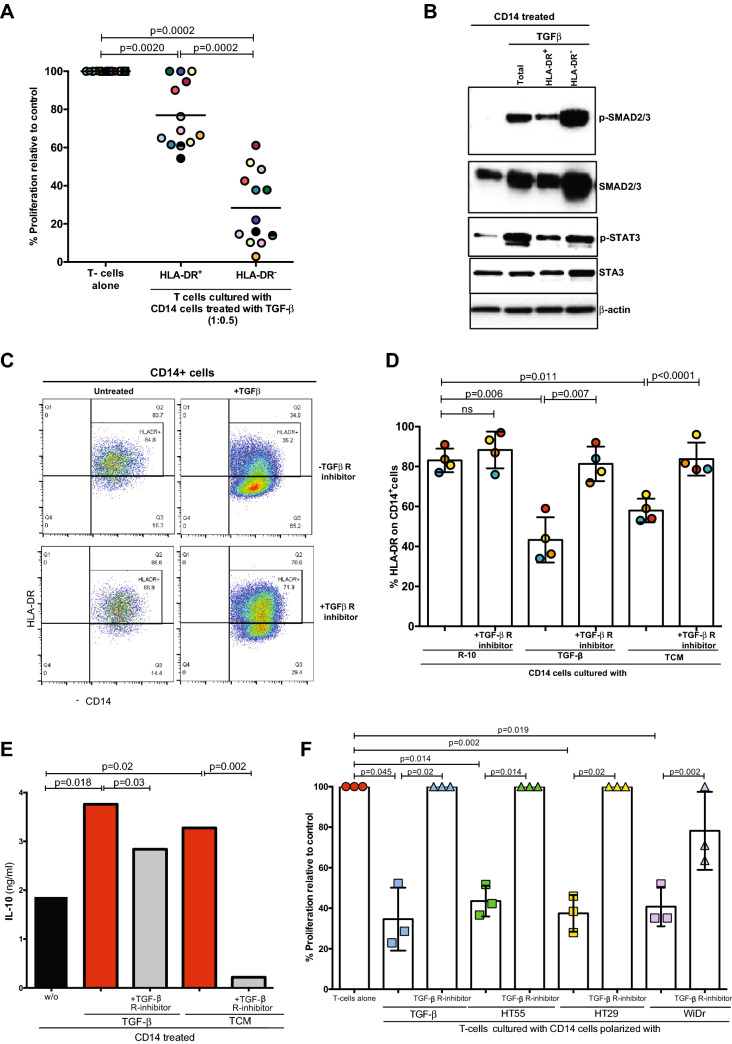
TGF-β derived M-MDSC suppress T cell proliferation. **a** TGF-β polarized CD14+HLA-DRcells suppress T-cell proliferation. Less suppressive activity has shown from TGF-β polarized CD14+HLA-DR+ cells. The 1:0.5 ratio of T cells: myeloid cells shown (n=13). **b** High expression of p-SMAD and p-STAT3 was detected by Western blot in the CD14+HLA-DR- cells polarized by TGF-β.Representative of 4 experiments. **c** and **d** TGF-β receptor inhibitor inhibits the ability of TGF-β and TCMs to down regulate HLA-DR on polarized monocytes (n=4). **e** TGF- β receptor inhibitor inhibits the release of IL-10 from TGF-β and TCM polarized monocytes. Representative of 2 experiments. **f**TGF- β receptor inhibitor inhibits the suppressive activity of CD14+HLA-DR- cells polarized with TGF-β and CRC cell TCM (n=3)

To identify the colorectal tumour factor(s) which mediates the polarization of M-MDSCs, tumour-conditioned media were analysed for cytokines by ELISA. A number of potential molecules were highly expressed including IL-10, G-GSF, IL-6, VEGF and TGF-β (Fig. 4c and Supp Fig. 4D) and these also accumulated in the plasma of CRC patients (Fig. 4d and Supp Fig. 4E). Culture of healthy donor monocytes with individual cytokines identified above showed that only TGF-β led to a significant down-regulation of HLA-DR (Fig. 4e, f and Supp Fig. 5A). The HLA-DR- fractions obtained following TGF-β treatment were associated with stronger immunosuppressive activity (Fig. 5a).

TGF-β superfamily ligands control many fundamental aspects of cellular behaviour including cell proliferation and survival. This cytokine binds to a type I and II receptor, expressed on different cancer cells and myeloid cells, initiating intracellular signals with phosphorylation of SMAD protein and subsequently phosphorylation of STAT3 to regulate cell migration and invasion [23]. To confirm the intracellular pathway regulated by TGF-β, healthy donors CD14^+^ cells were treated with TGF-β and subsequently the HLA-DR^−^ and HLA-DR^+^ fractions were enriched by sorting. No difference in the phosphorylation of p38 and ERK proteins were seen between HLA-DR^−^ and HLA-DR^+^ CD14 cells by Western blotting (Supp Fig. 5B). However, TGF-β treatment increased the phosphorylation of SMAD and STAT3 protein, particularly in HLA-DR^−^ cells (Fig. 5b). Treatment of CD14 + cells with TGF-β induced IL-10 release (Supp Fig. 5C). Finally, blockade with an anti-TGF-β receptor inhibitor interfered with the HLA-DR down-regulation on CD14^+^ cells treated with TGF-β and TCMs (Fig. 5c, d, Supp Fig. 5D), with IL10 release (Fig. 5e), and with suppressive activity (Fig. 5f, Supp Fig. 5E). Thus, TGF-β released by CRC cells causes activation of STAT3 and drives the production of M-MDSCs.

## Discussion

We show here that M-MDSCs in CRC tissue and in the blood of CRC patients strongly suppress T cell proliferation via the production of IL-10 and that TGF-β released by CRC cells is responsible for the polarisation of monocytes to become HLA-DR^−^ suppressors. We show that TGF-β treatment increases CD14^+^ pSTAT3 particularly in HLA-DR^−^ cells. STAT3 mediates down-regulation of MHC class II on antigen presenting cells [24, 25] and IL-10 production by macrophages [25] Previous work shows that suppressive M-MDSCs in pancreatic cancer have down-regulation of miRNAs that bind and repress the STAT3 promoter, have enhanced expression of pSTAT3 and that STAT3 inhibition abrogates CD14 + suppressivity [26] CRC M-MDSCs are mainly localised at the invasive margin of the cancer in line with the monocyte and TGF-β enrichment characteristic of consensus molecular subtype 4, the molecular subtype associated with the invasive front [27, 28].

Our novel mechanistic findings describing the cross-talk between colorectal cancer cells, monocytes and T cells in the tumour microenvironment are supported by previous studies in other clinical and pre-clinical contexts analysing the impact of TGF-β on monocytes and the role of monocyte-derived IL-10 in mediating immunosuppression. IL-10 directly inhibits T cell proliferation [29]. Stimulated monocytes produce IL-10 [30] and monocyte-derived IL-10 suppresses both CD3-stimulated and alloantigen-stimulated T cell proliferation and potently reduces IFNγ production [31], a cytokine essential for the activity of anti-PD1 therapy [32]. IL-10 transcripts are present in CD14^+^ leukocytes in the malignant ascites of ovarian cancer patients but not in the CD14^−^ fraction and are only present in the HLA-DR^−^ and not the HLA-DR^+^ CD14^+^ population [33]. Whilst T cells co-cultured with autologous CD14^+^ HLA-DR ^+^ cells produced IFNγ, no IFNγ was produced when T cells were co-cultured with CD14^+^ HLA-DR^−^ cells and the suppression of cytokine production could be reversed with IL-10 blockade. Co-incubation of autologous T cells with IL-10 producing monocytes inhibited their PHA-induced proliferation, and this inhibition could be reversed by the combination of anti-IL10R and anti-TGF-β which was also found to be specifically expressed in the IL-10 producing CD14^+^ cells. In the EL-4 tumour model IL-10 produced by macrophages increases in ascitic fluid in line with progression of disease and correlates with increased TGF-β produced by the cancer cells [34]. There is a significant reduction in peritoneal IL-10 upon administration of anti-TGF-β. In vitro the enhanced IL-10 production by macrophages was abrogated by the administration of anti-TGF-β.

Recent data interrogating the TCGA show upregulation of TGF-β1 in a number of cancers including CRC and this correlates with the markers expressed on M-MDSCs, CD14 and CD33, but not with the G-MDSC marker CD66b [35] (PMN-MDSCs are CD33^dim^ rather than the high expression seen on M-MDSCs [36]). TGF-β activity co-localised with the presence of CD11b^+^ cells [35]. TGF-β maintains CD124 expression and enhances the viability of healthy donor CD14^+^ monocytes. The addition of TGF-β to GM-CSF and IL-6 to monocyte cultures increases the proportion of HLA-DR^−^ cells consistent with polarisation to an M-MDSC phenotype, enhances suppression of CD8^+^ T cell proliferation and function, decreases macrophage differentiation and increases the production of anti-inflammatory cytokines. TGF-β blockade significantly reduces M-MDSC polarisation and increases the proportion of macrophage and HLA-DR^+^ DCs.

Our results are immediately translatable to the clinic. Given the fact that M-MDSCs appear to be the predominant MDSC population in CRC [21], that increase with treatment suggestive of a role in resistance [21] and are strongly immunosuppressive (data herein), abrogation of M-MDSC function may be a valuable adjunctive therapy both to immunotherapy and other standard of care therapies in CRC. Furthermore, given data demonstrating that M-MDSCs are the precursor of immune-suppressive TAMs [20], targeting of TAMs could be achieved by targeting the precursor M-MDSCs. We have recently reported on the impact of CD33 targeted therapeutics on MDSCs [37]. We confirmed that CD33 intensity was greater on M-MDSCs compared to PMN-MDSCs. Gemtuzumab ozogamicin (GO) is an antibody drug conjugate where the warhead calicheamicin is coupled to anti-CD33. We demonstrated that GO bound to patient CD33^+^ MDSCs and was rapidly internalised resulting in a dose-dependent decrease in viability, induction of apoptosis and the abrogation of the suppression of T cell proliferation [37].

The suppression of CAR-T proliferation by MDSCs against various targets was overcome by GO. We are about to initiate the GOTHAM trial of GO in solid cancers (principally CRC), HLH and MAS with a key focus on the impact of GO on the number and function of MDSCs. Given the essential role of M-MDSCs in immunosuppression and potentially in therapy resistance in CRC evaluation of its potential as an adjunctive therapy in CRC appears warranted.

### Supplementary Information

Below is the link to the electronic supplementary material.

Phenotypic analysis of myeloid cells from blood and tissue of CRC patients. (**A**) Similar expression of CD33 was measured on CD14^+^ cells from the blood (n=34) and the tumour tissue (n=31) of colorectal patients compared healthy donors (n=19) and normal tissue (n=10). (**B**) No correlation was observed between the frequency of CD14^+^HLA-Dr^-^ and CD14^+^HLADr^+^ cells of CRC patients’ tissue and blood samples, linear correlation line shown (PDF 57 KB)

Tumour localization of CD14^+^ myeloid cells in colorectal cancer. Multispectral immunohistochemical staining of 5 colorectal cancer section. CD14: Yellow; HLA-DR: Magenta; CD3: Cyan/green; CK: red; nuclei: Blue (PDF 181 KB)

Dose-dependent immunosuppressive activity of CD14^+^HLA-DR^-^ cells. Allogeneic T cell proliferation under CD3/CD28 antibody stimulation is suppressed with the addition of increasing number of CD14^+^HLA-DR^-^ cells from the tissue (**A**) and the blood (**B**) of CRC patients, as measured by 3H-Tymidine uptake. The CD14^+^HLA-DR^+^ population is not suppressive. (**C**) The addition of T cell proliferation of arginase and iNOS inhibitors (NOHA and L-NMMA) didn’t inhibit the suppressive activity of CRC patient derived CD14^+^ cells (PDF 42 KB)

HLA-DR expression and IL-10 release by monocytes treated with colorectal cancer cell line TCM. (**A**) Colorectal cell lines down regulate HLA-DR on CD14^+^ cells. Each colour represents CD14^+^ cells from different healthy donors. (**B**) Higher release of IL-10 was measured by CD14^+^HLA-DR^-^ cells polarized with colorectal cell lines. ELISA shows IL-10 measured in the supernatant of CD14^+^ cells treated with three different colorectal cell lines. (**C**) TCMs were analysed for cytokines with a LEGENDplex beads-based assay (n=17). (**D**) Higher concentration of TGF-β was measured by ELISA in TCM of colorectal cell lines (3 independent experiments). (*E*) Plasma of CRC patients were analysed for cytokines with a LEGENDplex beads-based assay (n=39) (PDF 521 KB)

Cytokines analysis of TCM and CRC patient plasma. (**A**) HLA-DR down regulation was analysed by flow cytometer on TGF-β polarized CD14+ cells. B) No change in the phosphorylation of p38 and ERK1/2 was detected by Western blot in the CD14^+^HLA-DR^-^ and CD14^+^HLA-DR^+^ cells polarized by TGF-β. (**C**) Significantly higher level of IL-10 was detected by ELISA in the supernatant of CD14^+^ cells treated with TGF-β (10μg/ml). (**D**) TGF-β receptor inhibitor inhibits the downregulation of HLA-DR on CD14^+^ cells polarized with TGF-β and TCM from colorectal cell lines (n=3). (**E**) TGF-βreceptor inhibitor inhibits the dose-dependent suppressive activity of CD14^+^HLA-DR^-^ cells polarized with TGF-β (n=6) (PDF 3949 KB)

## Data Availability

Source data will be made available in the event of acceptance for publication.
