# Supplementary figures and images for: TGF-β orchestrates the phenotype and function of monocytic myeloid-derived suppressor cells in colorectal cancer
Source: Cancer Immunol Immunother. 2021 Nov 2;71(7):1583–96. doi: 10.1007/s00262-021-03081-5 (PMC9188538; doi:10.1007/s00262-021-03081-5)

A

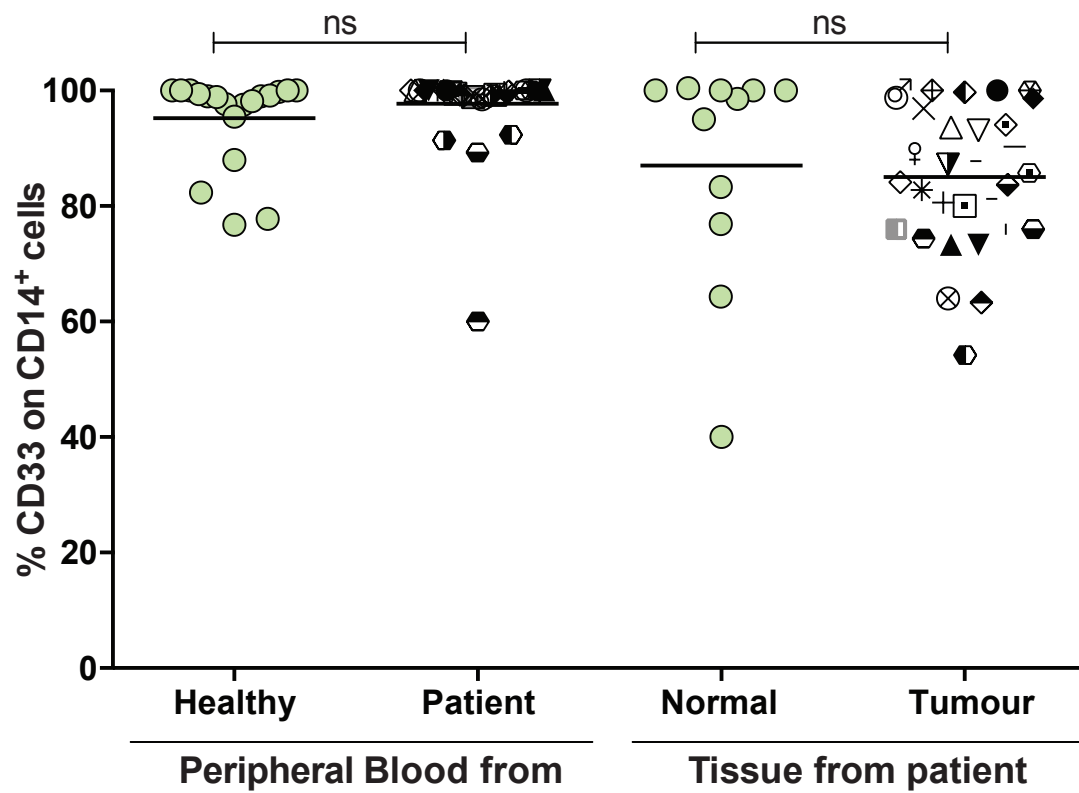

B

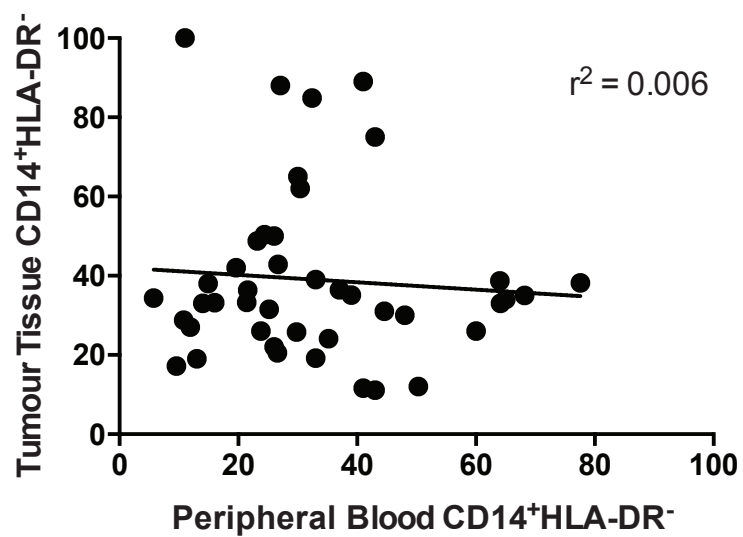

Supplement: Supplementary file 1 — Phenotypic analysis of myeloid cells from blood and tissue of CRC patients. (A) Similar expression of CD33 was measured on CD14+ cells from the blood (n=34) and the tumour tissue (n=31) of colorectal patients compared healthy donors (n=19) and normal tissue (n=10). (B) No correlation was observed between the frequency of CD14+HLA-Dr- and CD14+HLADr+ cells of CRC patients’ tissue and blood samples, linear correlation line shown (PDF 57 KB) [file 262_2021_3081_MOESM1_ESM.pdf]

## INVASIVE MARGIN

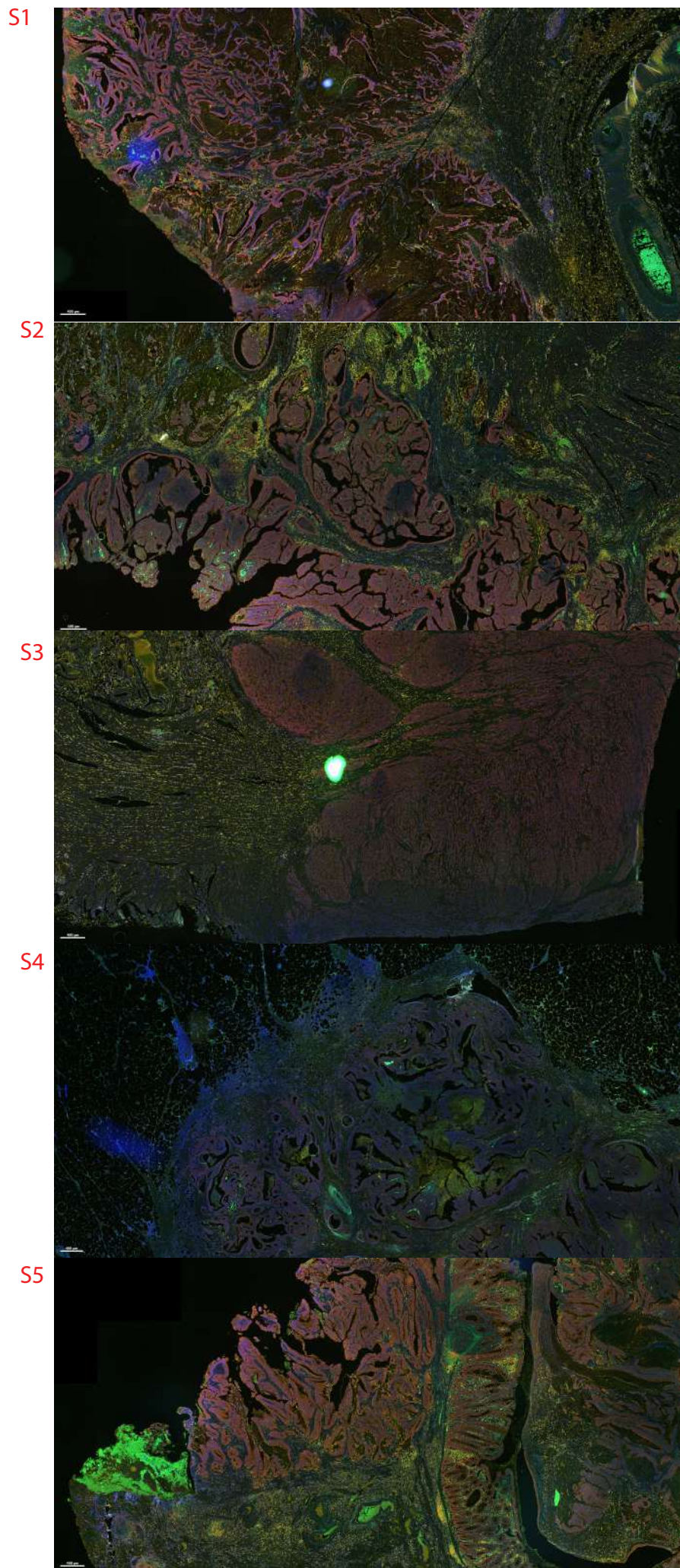

Supplement: Supplementary file 2 — Tumour localization of CD14+ myeloid cells in colorectal cancer. Multispectral immunohistochemical staining of 5 colorectal cancer section. CD14: Yellow; HLA-DR: Magenta; CD3: Cyan/green; CK: red; nuclei: Blue (PDF 181 KB) [file 262_2021_3081_MOESM2_ESM.pdf]

A

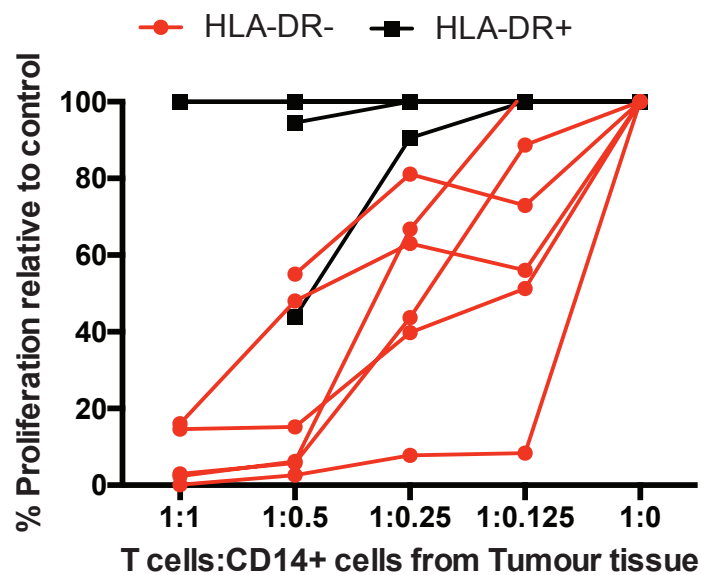

B

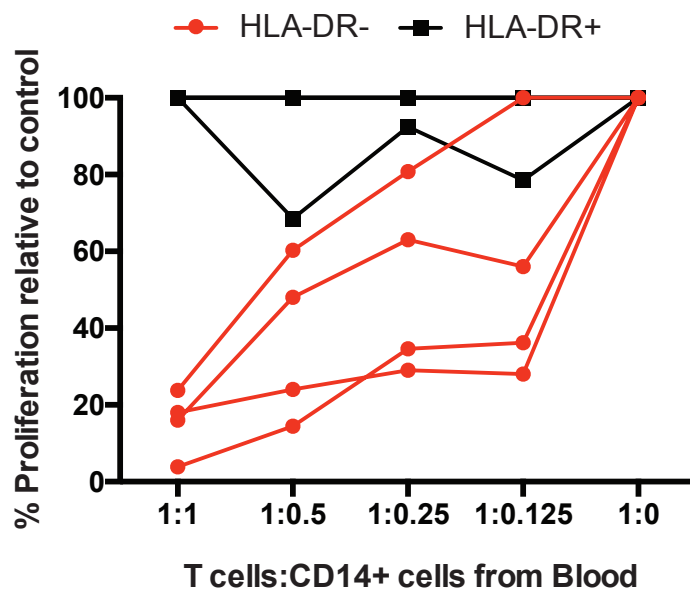

C

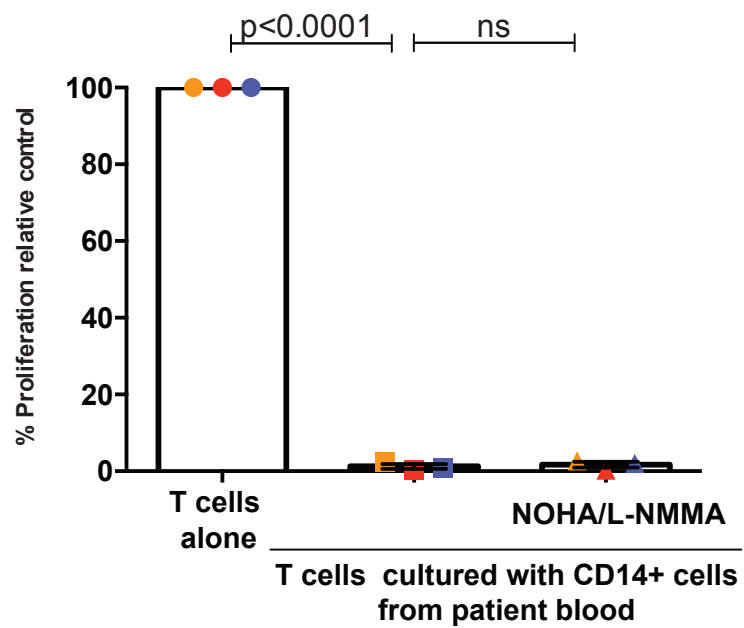

Supplement: Supplementary file 3 — Dose-dependent immunosuppressive activity of CD14+HLA-DR- cells. Allogeneic T cell proliferation under CD3/CD28 antibody stimulation is suppressed with the addition of increasing number of CD14+HLA-DR- cells from the tissue (A) and the blood (B) of CRC patients, as measured by 3H-Tymidine uptake. The CD14+HLA-DR+ population is not suppressive. (C) The addition of T cell proliferation of arginase and iNOS inhibitors (NOHA and L-NMMA) didn’t inhibit the suppressive activity of CRC patient derived CD14+ cells (PDF 42 KB) [file 262_2021_3081_MOESM3_ESM.pdf]

A

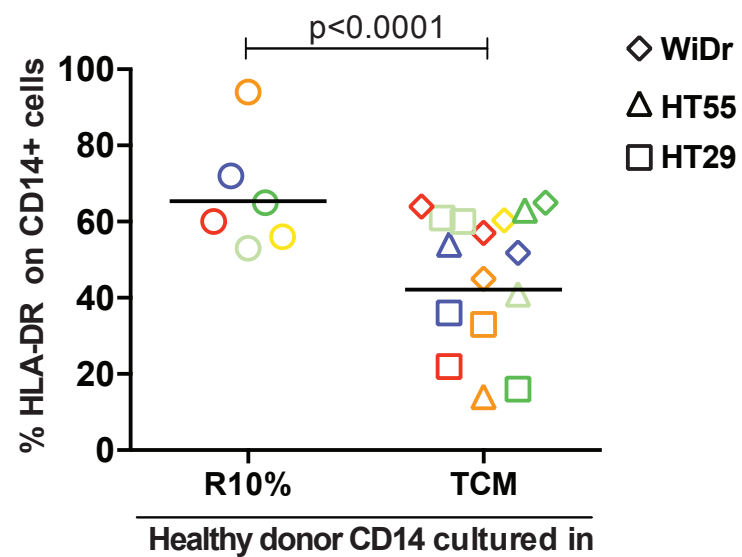

B

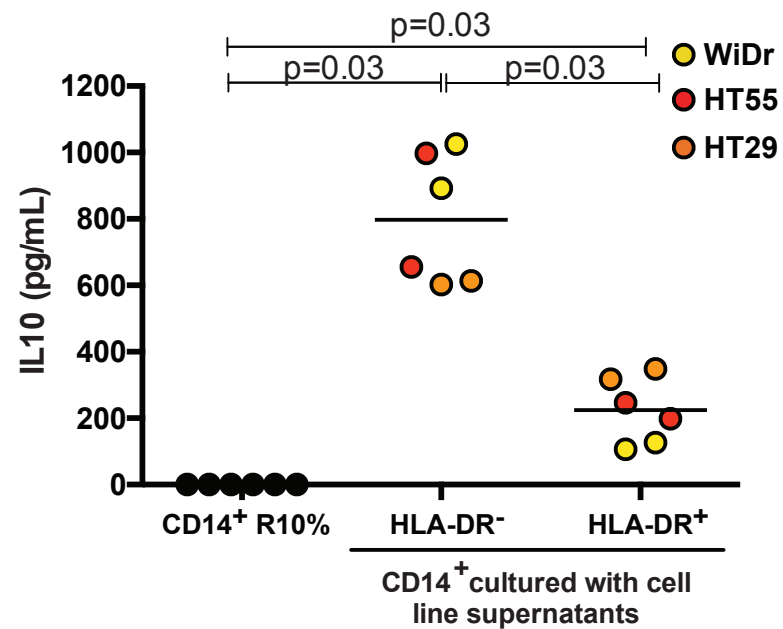

C

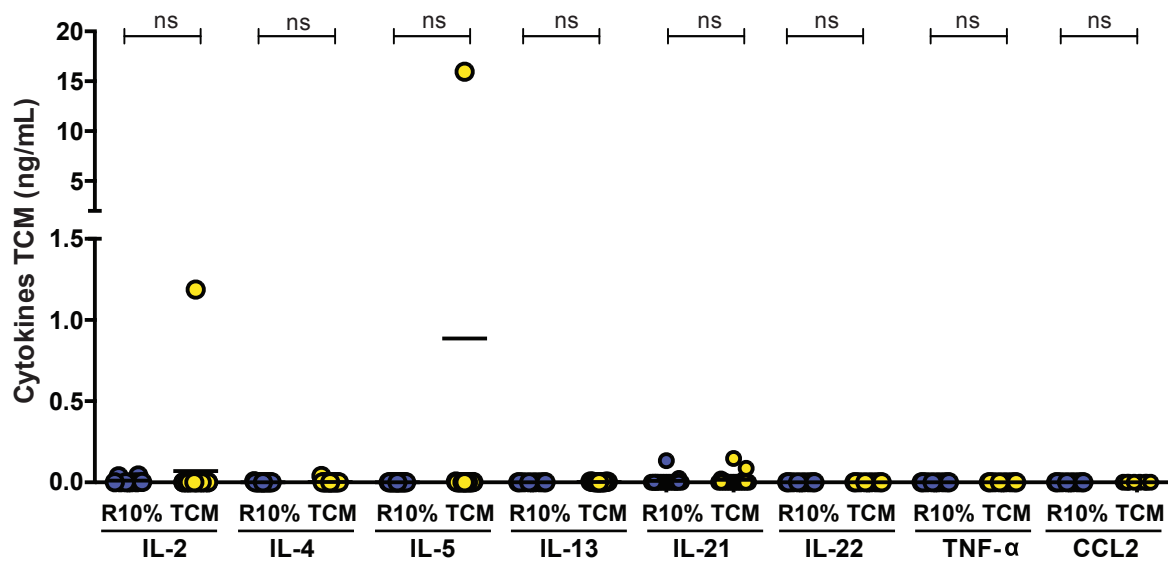

D

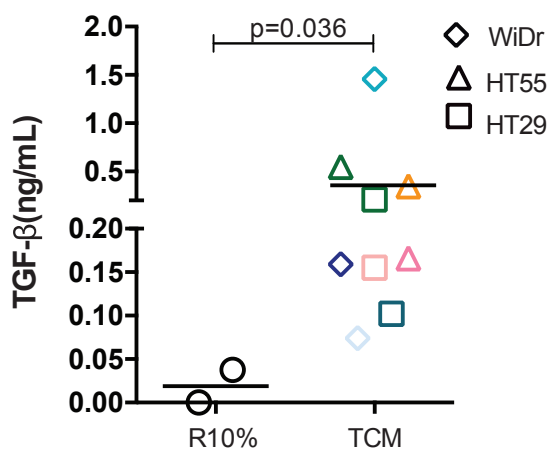

E

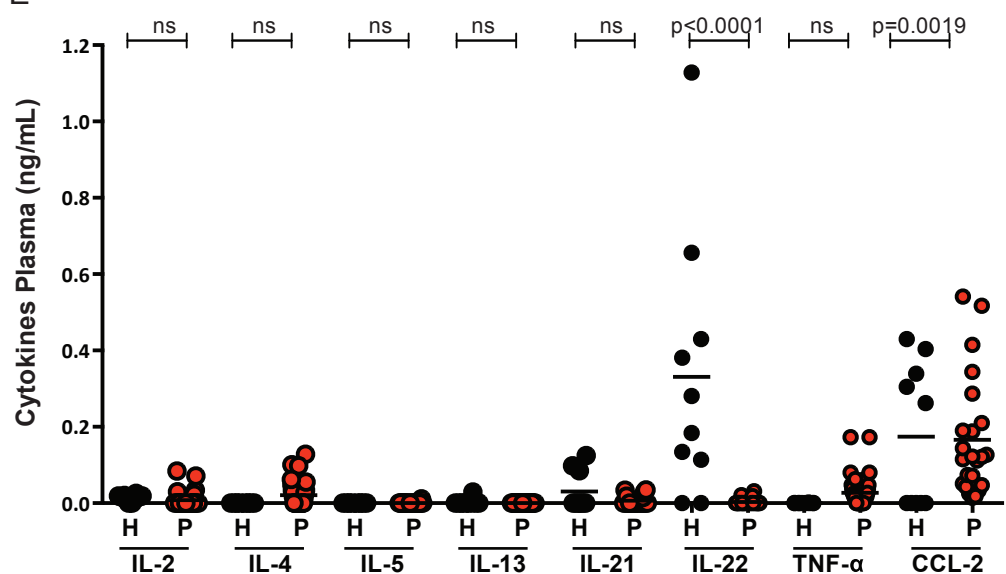

Supplement: Supplementary file 4 — HLA-DR expression and IL-10 release by monocytes treated with colorectal cancer cell line TCM. (A) Colorectal cell lines down regulate HLA-DR on CD14+ cells. Each colour represents CD14+ cells from different healthy donors. (B) Higher release of IL-10 was measured by CD14+HLA-DR- cells polarized with colorectal cell lines. ELISA shows IL-10 measured in the supernatant of CD14+ cells treated with three different colorectal cell lines. (C) TCMs were analysed for cytokines with a LEGENDplex beads-based assay (n=17). (D) Higher concentration of TGF-β was measured by ELISA in TCM of colorectal cell lines (3 independent experiments). (E) Plasma of CRC patients were analysed for cytokines with a LEGENDplex beads-based assay (n=39) (PDF 521 KB) [file 262_2021_3081_MOESM4_ESM.pdf]

A

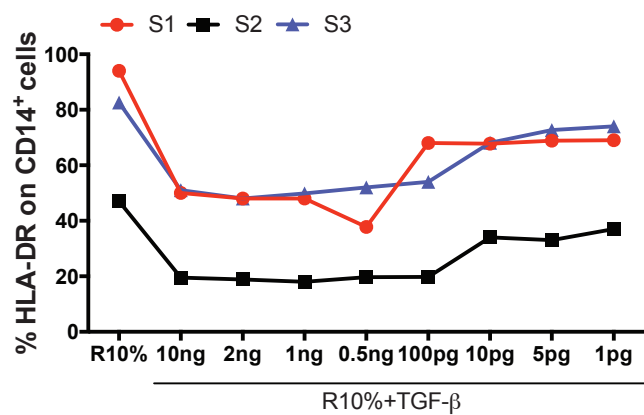

B

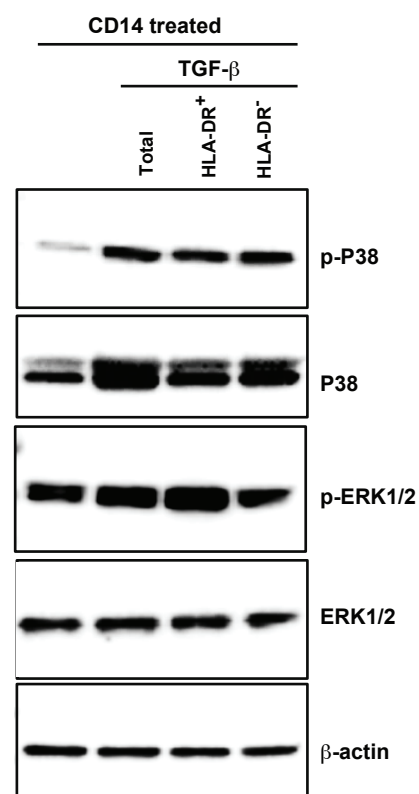

C

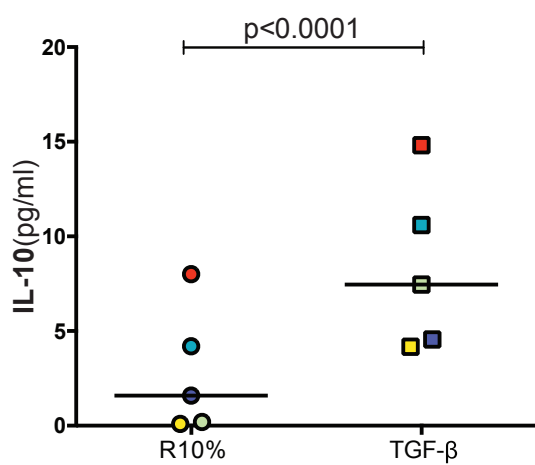

D

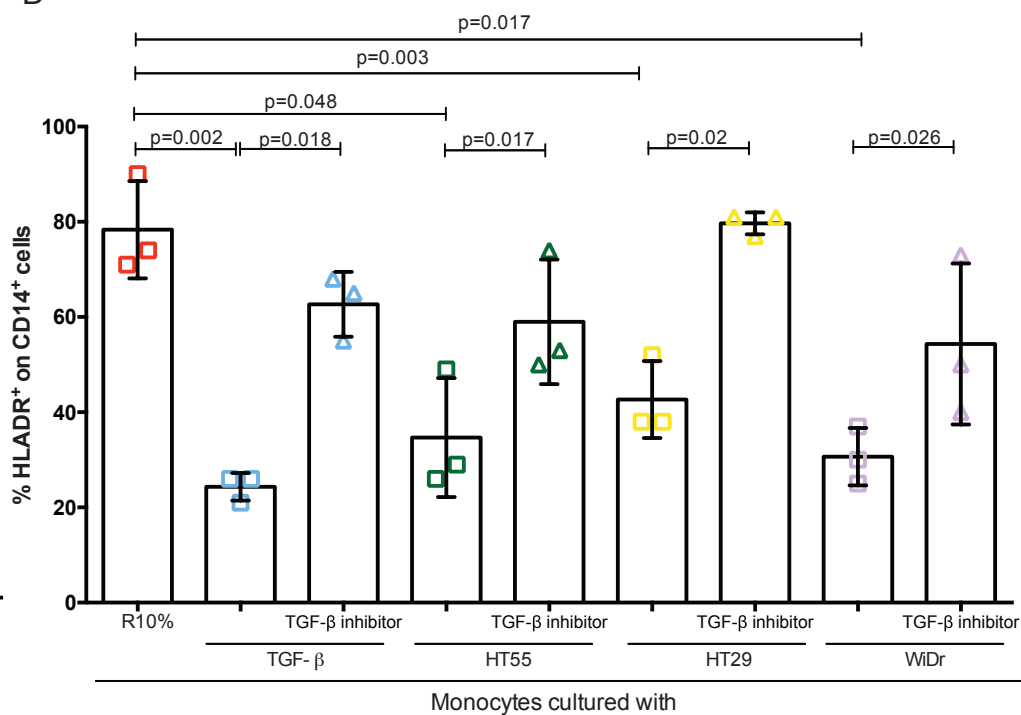

E

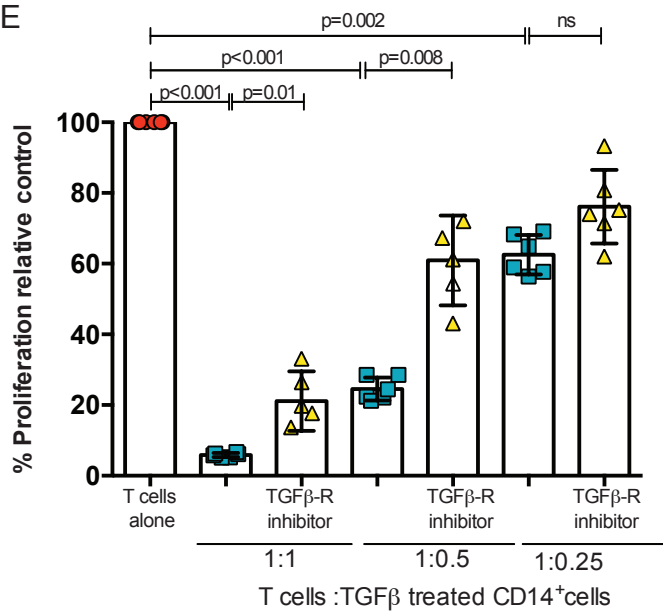

Supplement: Supplementary file 5 — Cytokines analysis of TCM and CRC patient plasma. (A) HLA-DR down regulation was analysed by flow cytometer on TGF-β polarized CD14+ cells. B) No change in the phosphorylation of p38 and ERK1/2 was detected by Western blot in the CD14+HLA-DR- and CD14+HLA-DR+ cells polarized by TGF-β. (C) Significantly higher level of IL-10 was detected by ELISA in the supernatant of CD14+ cells treated with TGF-β (10μg/ml). (D) TGF-β receptor inhibitor inhibits the downregulation of HLA-DR on CD14+ cells polarized with TGF-β and TCM from colorectal cell lines (n=3). (E) TGF-βreceptor inhibitor inhibits the dose-dependent suppressive activity of CD14+HLA-DR- cells polarized with TGF-β (n=6) (PDF 3949 KB) [file 262_2021_3081_MOESM5_ESM.pdf]
